# More evidence is needed for the association between serum myasthenia gravis and adverse pregnancy, delivery, and neonatal outcomes

**DOI:** 10.1111/ene.16161

**Published:** 2023-11-20

**Authors:** Yi‐Ping Song, Liang Ma

**Affiliations:** ^1^ Kangda College of Nanjing Medical University Lianyungang Jiangsu China; ^2^ Lianyungang No. 1 People's Hospital Lianyungang Jiangsu China

Dear Editor,

We read with the great interest the article titled "Pregnancy Outcomes for Women With Myasthenia Gravis and Their Newborns: A Nationwide Register‐Based Cohort Study" by Laura O'Connor et al. [1]. The authors found that pregnancy complications did not show an increased risk in patients with myasthenia gravis (MG). The authors have made valuable contributions to the existing literature, but there are several crucial aspects that require further deliberation.

First, the study failed to address important confounders. It has been suggested that factors such as marital status, infant sex, and monthly household income may serve as potential confounders [2], particularly considering the association of anemia with adverse pregnancy outcomes [3]. However, the authors did not incorporate them as confounding variables in the logistic regression model, which could potentially impact the adjusted odds ratio of MG and outcomes, thereby influencing the determination of any association between these factors.

Furthermore, there are certain limitations associated with the logistic regression approach employed in this study. (i) The criteria employed by the authors for variable selection remain undisclosed. In the case of multiple regression models, there typically exist two approaches to variable selection. First, variables were chosen based on the statistical outcomes of univariate analysis. Mickey and Greenland proposed employing a significance level of 0.25 as a threshold for selecting independent variables in logistic regression models [4]. Second, variables were selected through stepwise regression analysis. The stepwise regression analysis involves initially conducting a simple regression for each explanatory variable, followed by the gradual introduction of other explanatory variables that contribute significantly to the explained variable [5]. We recommend authors provide a comprehensive account of the methodologies and criteria employed for variable selection. (ii) The authors did not report pseudo‐*R* ^2^s. *R* ^2^ is used to assess goodness of fit. It represents the proportion of variance in the criterion that is explained by the predictors [6]. We suggest reporting pseudo‐*R* ^2^ to test whether the risk factors included are comprehensive. If the Cox and Snell *R* ^2^ and Nagelkerke *R* ^2^ value is too small, the result may be that the authors miss some important risk factors as covariables.

In addition, the administration of medication in patients with MG may exert a certain influence on the neonatal outcome. The administration of azathioprine has been associated with potential adverse effects on fetal growth and concerns regarding immune alterations [7]. Mycophenolate mofetil has been reclassified as a class D drug by the US Food and Drug Administration due to documented teratogenic effects on human fetuses [8]. Therefore, we propose conducting separate binary logistic regression analyses for MG patients receiving drug treatment to investigate the potential impact of medication on neonatal outcomes, which will enhance the overall rigor of their study.

Their findings provide further evidence supporting the absence of significant adverse effects of MG on pregnancy outcomes. However, we contend that additional empirical evidence is imperative to substantiate the stated conclusions. We cordially invite the authors to provide further elucidation on their research findings.

## AUTHOR CONTRIBUTIONS

**Liang Ma:** Supervision. **Yi‐Ping Song:** Writing – original draft.

## CONFLICT OF INTEREST STATEMENT

Neither of the authors has any conflict of interest to disclose.

## Data Availability

Data availability is not applicable to this article as no new data were created or analyzed in this study.
